# Hand Washing Gesture Recognition Using Synthetic Dataset

**DOI:** 10.3390/jimaging11070208

**Published:** 2025-06-22

**Authors:** Rüstem Özakar, Eyüp Gedikli

**Affiliations:** 1 Deparment of Computer Engineering, Faculty of Engineering and Architecture, Erzurum Technical University, Erzurum 25100, Turkey; 2 Deparment of Computer Engineering, Faculty of Computer and Information Sciences, Trabzon University, Trabzon 61300, Turkey; eyupgedikli@trabzon.edu.tr

**Keywords:** computer vision, machine learning, hand washing, hand gesture recognition, synthetic dataset, rendering

## Abstract

Hand hygiene is paramount for public health, especially in critical sectors like healthcare and the food industry. Ensuring compliance with recommended hand washing gestures is vital, necessitating autonomous evaluation systems leveraging machine learning techniques. However, the scarcity of comprehensive datasets poses a significant challenge. This study addresses this issue by presenting an open synthetic hand washing dataset, created using 3D computer-generated imagery, comprising 96,000 frames (equivalent to 64 min of footage), encompassing eight gestures performed by four characters in four diverse environments. This synthetic dataset includes RGB images, depth/isolated depth images and hand mask images. Using this dataset, four neural network models, Inception-V3, Yolo-8n, Yolo-8n segmentation and PointNet, were trained for gesture classification. The models were subsequently evaluated on a large real-world hand washing dataset, demonstrating successful classification accuracies of 56.9% for Inception-V3, 76.3% for Yolo-8n and 79.3% for Yolo-8n segmentation. These findings underscore the effectiveness of synthetic data in training machine learning models for hand washing gesture recognition.

## 1. Introduction

Almost all interactions of people with their surroundings happen through hands; for this reason, hands have to be frequently washed using water to clean off viruses and bacteria. Performing hand washing in compliance with the procedures for disinfection carries great importance, especially in the health sector. For this purpose, special hand washing gestures are recommended by the World Health Organization [1]. Incorrect and inadequate hand washing can result in infections, supervising the procedure would benefit public health considerably. A significant risk of mortality, healthcare-associated infections (HAIs), are primarily caused by healthcare workers. A systematic review concluded that hand hygiene compliance reduces the HAIs [2].

Monitoring hand hygiene with human supervision can be impractical for several reasons. Frequent inspections may be burdensome and objectivity can also be an issue. With the help of autonomous systems, these problems can be mitigated. Studies showed that using monitoring devices for hand hygiene compliance were beneficial [3].

There are various approaches using machine learning to autonomously inspect hand washing, mostly making use of computer vision systems or various devices with sensors. After the COVID-19 pandemic, non-invasive autonomous systems gained importance for inspecting the hand washing procedures of persons, especially in the food and health sectors. In this context, hand washing evaluation became an extensive research topic in computer vision.

This study addresses a significant issue in this research area: the lack of comprehensive datasets. In addition to very few datasets and no established standards regarding hand washing research, there are also difficulties with creating a comprehensive dataset. A dataset in hand washing requires several participants to wash their hands in different places for long durations, which can be physically demanding and time consuming. Furthermore, with mounting cameras in bathrooms, obtaining permissions can be an issue. To overcome these issues, synthetic data can be created using computer-generated imagery (CGI), which is already very advanced nowadays.

Using CGI to create a dataset, especially for hand gesture problems, can be more beneficial than using other methods such as generative neural network models, because hand gestures need to be represented precisely in order to have clear distinction between gestures and successful model training. Generative models may not have sufficient data for this particular problem to produce reliable spatio-temporal data. In this work, a synthetic dataset consisting of different environments and characters has been created and its success in gesture classification was tested in a real-world hand washing dataset using different neural network models.

This article has the following sections: in the literature review, there is a brief overview of recent studies on hand washing and synthetic data are given. In the Materials and Methods Section, the design and features of the synthetic dataset and real-world test dataset are explained in detail. Neural network models, training and the parameters are also explained. In the Results Section, the test results are examined with confusion matrices and other metrics. In the Discussion Section, the results and possible future works are discussed.

## 2. Literature Review

Hand washing gesture recognition is essentially a hand gesture recognition problem. There has been many approaches for hand gesture recognition in the literature, however, due to the success of deep learning networks in recent years, they became the most common approach. For classifying hand washing gestures, studies in the literature can be categorized into two main categories: sensor-based or vision-based. Generally, studies focus mainly on gesture classification or genuineness of washing with the help of machine learning.

Current vision-based methods have the drawbacks of limited datasets and a lack of hand washing gesture standardization. Sensor-based models have the main drawback of making the participant wear a device with sensors, which may not be suitable or comfortable for all situations. In both categories, studies showed that models are able to achieve good performance. However, without standardization and benchmarking datasets, comparative evaluation of the proposed methods is not possible. In addition to the literature review in our previous work [4], some recent studies can be summed as follows:

Hagpanah et al. [5] conducted a study for evaluating the quality of hand washing. Ju et al. [6] created a synthetic dataset similar to this study in their work to research the effects of cast shadows on hands while hand washing. Lattanzi et al. [7] aimed to optimize machine learning models that are used with wearable sensors in order to reduce energy cost. Asif et al. [8] developed a Residual-Based Multilevel Fused Network architecture, which uses different networks together, and applied it to two different datasets. Pepito et al. [9] used Yolo-v5 (You Only Look Once) and Yolo-v8 models to detect mask, hairnet and hand washing gestures using a camera mounted above the basin.

There are very few datasets available for hand washing. Studies that present a custom dataset can be seen in [10,11,12]. These datasets do not have commonly defined gestures or camera positions. Common gesture sets and camera positions should be established to standardize hand washing studies and contribute to the creation of new datasets.

Synthetic datasets are commonly used in various research fields in machine learning to solve real-world problems. Synthetic data can be generated using generative machine learning models or computer-generated imagery. A comprehensive literature review of these datasets and used methods can be found in [13,14].

## 3. Materials and Methods

To perform vision-based hand washing gesture recognition, frame-based classification using CNNs (Convolutional Neural Networks) and object-based detection using Yolo [15] networks can be used. Each frame of a gesture can be cropped to have hand regions and can be used for training CNNs, or hands from each gesture can be considered objects in each frame and object detection with Yolo can be performed. Furthermore, depth images can be used to create point clouds and classification with networks like PointNet (a point cloud processing network) becomes possible. In this study, the frame-based classification goal was achieved using these neural networks using synthetic data.

### 3.1. Synthetic Dataset

In a hand washing evaluation, it is important to design a system that does not constrain the person performing the process. Furthermore, because of the water presence, the system should be protected from water damage. Although common and successful, sensor-based approaches may not be comfortable enough for the participant because they need to be worn during hand washing. Sensor-based devices also need to be waterproof because they can easily come into contact with water. Considering these points, vision-based systems can be considered more robust for hand washing evaluation. A camera stationed above the participant with enough distance would be the ideal solution, so the camera would be able to capture both hands and any water contact would be prevented. Furthermore, participants can perform hand washing unrestrictedly.

**Figure 1:**
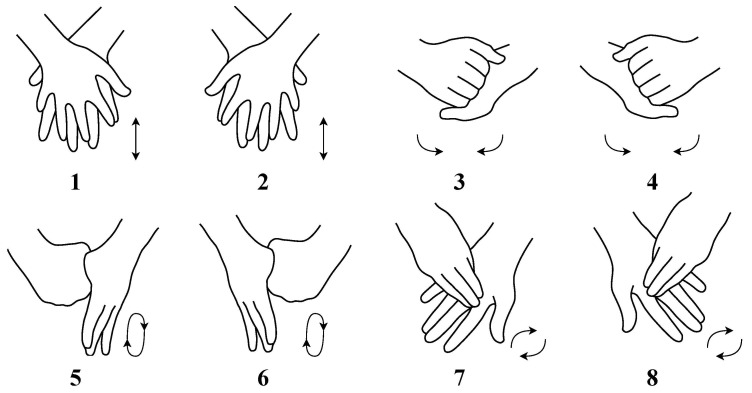
Eight hand washing gestures used in this dataset [4].

In this research, an animated synthetic dataset was created containing eight different hand washing gestures, using four characters in four environments. Gestures can be seen in Figure 1. For each gesture, two different animations per character were prepared for each environment. For each gesture, basis animations were prepared using keyframes without motion capture with linear interpolation, and these animations were then applied to different characters with variations to prevent repetitiveness. For each gesture, four different environment sessions were prepared, each session containing four characters performing the gesture, with two different animations. Thus, 8 × 4 × 4 × 2 = 256 varied animations were created. Each session contains 375 frames, rendered with 25 frames per second corresponding to 15 s of animation. For each gesture, 12,000 frames were created. In total 96,000 frames were created for the dataset. Each gesture contained 750 frames per environment, totaling 3000 frames per gesture. Each environment had 6000 frames in total for all gestures. The dataset was equivalent to 64 min of content.

**Figure 2:**
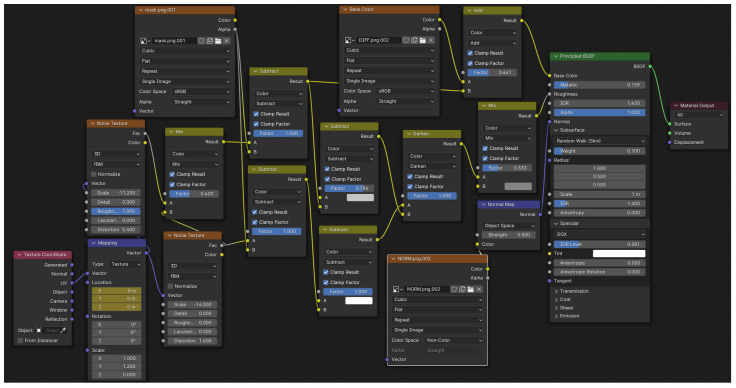
Node-based material shader in Blender for giving procedural soapy/wet hand look.

Each environment was designed to have different styles, lighting and materials. Cameras were placed at varied distances from the basins in each environment. Characters were designed to have different physical appearances, hand anatomies and skin tones. One of the two animation sessions for each gesture was designed to have a moist/wet hand look, and the other was designed to have a procedural soapy look, which used a material shader created with a node system (Figure 2). The above implementations were made to achieve variety in the dataset.

To create rendered images, a free-to-use open source software, Blender 4.1 [16], was used. Environments were modeled without using a premade model. Textures were obtained from unrestricted free-to-use resources. Characters were modeled using another free-to-use open source software Makehuman [17] using parameters and default clothes. Textures of two characters were obtained from [18,19]; the other two characters’ default textures were used with modifications. The hand model of one of the male characters was obtained from [20].

**Figure 3:**
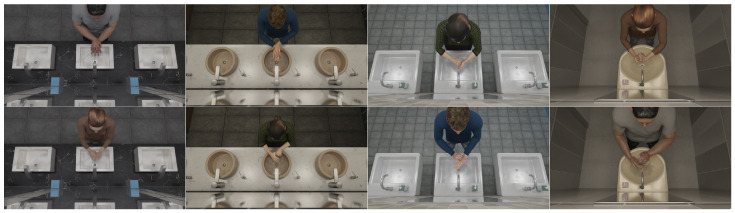
Examples from dataset showing different characters performing eight different gestures in four environments.

**Figure 4:**
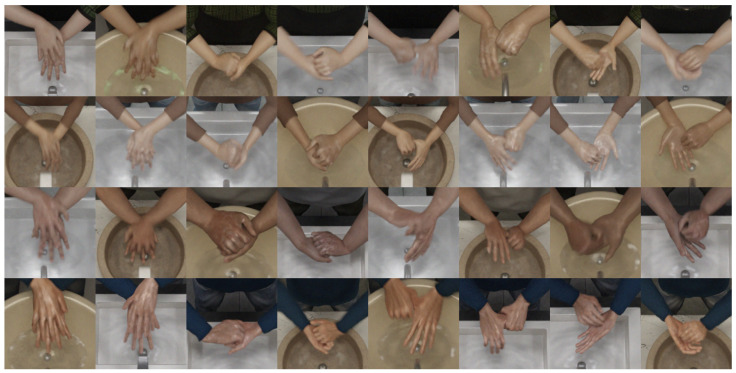
Region of interest (ROI) images from synthetic dataset showing eight different gestures performed by four different characters. Procedural soap amounts/wet look and motion blur can be seen.

**Figure 5:**
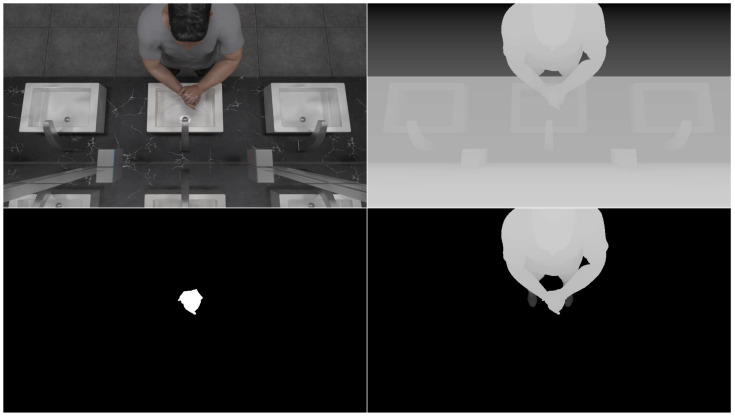
RGB (raw color), depth (distance data), binary mask (hand segmentation) and isolated depth (person-only distance) images within the dataset.

In addition to regular RGB images, depth, isolated depth and binary hand mask images were also created in the dataset to be used with point cloud and segmentation networks. To achieve the binary mask effect, a black color was used as texture for the characters, except for the hand regions, where a white color was used. Furthermore, hand material was set to be emissive using the white color, and all materials were set to be non-metallic and non-reflective in Blender. In Figure 3 and Figure 4 are examples from the dataset, and in Figure 5, different types of image data can be seen. Depth images in Blender can be acquired using render settings. Original depth images needed to be color inverted and normalized using nodes in the compositing settings in Blender. Isolated depth images were created by rendering only the character on the scene during animation. All depth images were saved as 16-bit PNG files.

The path-tracing-based Cycles render engine of Blender was used for realistic rendering. Motion blur resulting from the movement was also simulated in Blender to increase realism. Furthermore, lens noise was added to each frame using compositing in Blender. Each frame was created in 960 × 540 pixels in order to make them suitable for computer vision and shorten the rendering times. In Blender, the render area can be specified. Inside this 960 × 540 frame was a vertical box containing all dynamic motion and shadow changes were determined and renders were created using this area. After completion of the renders, these areas were pasted into the background static renders of the environments using OpenCV [21]. Renders were taken using 64 maximum samples, with a noise threshold of 0.01 in Blender parameters. The max. light bounces parameter was set to 12. Generally other parameters were used with default values.

The cameras in all the environments were perspective cameras used with default setttings and 80 degrees of FoV (Field of View). Environment 1 had 12 area lights, all of them 0.25 in size. Five of them were disk-shaped with 3-watt power. Seven of them were square-shaped with 2-watt power. Environment 2 had 12 area lights, five of them were in an ellipse shape with a 0.1 × 0.25 size, with 4-watt power. Four of them were in a square shape with 2-watt power. Three of them were in a square shape with 10-watt power. Square-shaped lights had the size of 1. Environment 3 also had 12 area lights, all of them square. Five of them had 4-watt power with a size of 0.25. Four of them had 5-watt power with a size of 1. Three of them had 10-watt power with the size of 1. Environment 4 had a single area light in a disk shape with a size of 1, having 6-watt power. In render settings, light tree option was enabled, default light parameters were used, max bounce value was set to 12 and direct and indirect light values were set to 12.

Renders were created using three different computers simultaneously. System 1 specifications were Radeon RX 6800 16 GB GDDR6 GPU, AMD Ryzen 5 5600X CPU, 32 GB DDR4 RAM. System 2 specifications were Intel Core i5-940 CPU, 8 GB DDR4 RAM with no external GPU. System 3 specifications were Radeon RX 580 8 GB GDDR5 GPU, Intel core i3-810 CPU, 32 GB DDR4 RAM. GPU compute was used with system 1. Approximate frame rendering times for the environments in the three systems are given in Table 1.

### 3.2. Real-World Dataset

In a previous study [4], a dataset was created with two participants (30-year-old male and 21-year-old female) in two different bathrooms containing eight hand washing gestures performed for 400 sessions. Sessions contained varying washing speeds and soap amounts. The dataset was recorded with a Kinect 2 camera, which provides RGB and depth images and skeleton tracking capability. Depth cameras are used in varied research areas such as gesture recognition [22], healthcare [23], robotics [24] and many more.

**Figure 6:**
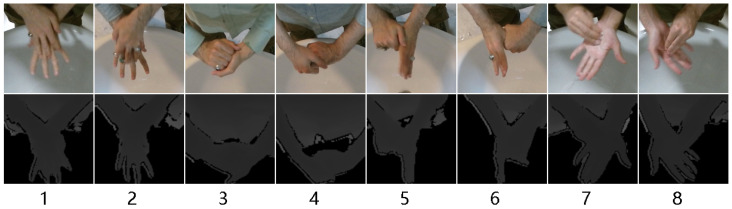
Example RGB and depth ROI frames from real-world dataset for each gesture (1–8) [4].

**Figure 7:**
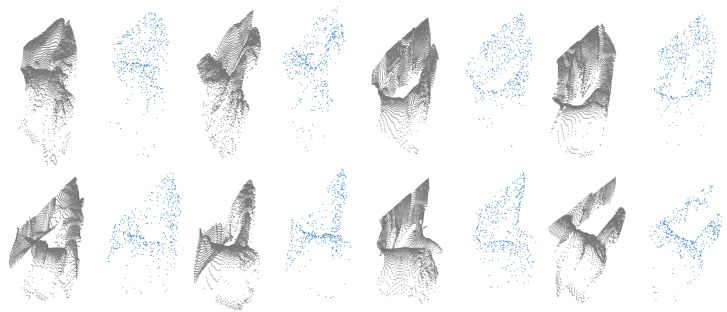
Example raw point cloud data (gray) and uniformly sampled 512 points (blue) for each gesture in real-world dataset [4].

The dataset consisted of 960 × 540 RGB, 150 × 150 RGB ROI (region of interest), 512 × 424 depth, 100 × 100 depth ROI images and point clouds created from depth files. In this dataset, hand regions were obtained using the tip point detection method after using background substraction on depth images. After background substraction, the participant performing the hand washing was isolated and the lowest y coordinate of the segmented person was considered as the tip point of the hands. In the Kinect 2 camera, depth and color images were aligned; a region of interest around this tip point of the depth image also matched to the color image coordinates, thus hand regions in color images were obtained. Examples from this dataset can be seen in Figure 6 and Figure 7. Frame amounts from this dataset for RGB images (same as RGB ROI) and point clouds can be seen in Table 2 for each gesture. This dataset was used as a test dataset for the models that were trained using the synthetic dataset. Further details about the dataset can be seen in [4].

### 3.3. Training Neural Networks with Synthetic Dataset

The synthetic dataset created in this study was used for training different neural networks and classifying gestures in real-world data. Neural networks from different architectures, Inception-V3 [25], Yolo-8n [15], Yolo-8n segmentation and PointNet [26], were selected and trained as classifiers. Inception-V3 is a widely used robust Convolutional Neural Network (CNN) model, which can perform image-based classifying. Its architecture makes use of asymmetrical convolutional filters. Yolo-8 is another widely used CNN architecture that can perform object detection. The network is trained using images that contain labeled bounding boxes for different classes. If a binary mask image of the object is provided, it can be trained using the contour information and the network can also perform segmentation. PointNet is a neural network used for point cloud classification/segmentation. Its architecture is similar to CNN networks, and its internal modules aims to make the point cloud order and transformation independent. PointNet and its derivatives are used in various research areas, from gesture recognition [27] to medical [28] and from autonomous driving [29] to robotics [30].

In mask images, the tip points of the hands were detected using the tip point detection method from previous study [4]. In this method, the lowest y coordinate pixel of the binary image is considered to be the tip point of the hands. Around this pixel a 150 × 150 ROI was cropped and resized to 96 × 96 for the Inception-V3 network. The inception model was trained for two epochs observing the accuracy and loss metrics. Since these metrics showed enough convergence, further training was not needed. Five-fold cross validation was used, determining theaverage accuracy, loss, validation accuracy and validation loss values for five different models can be seen in Table 3. For validation, 20% of the dataset was used. In network architecture, Dropout(0.3), Dense(64) with ReLU, Dropout(0.3) and Dense(8) with Softmax layers were used after the default Inception-V3 model. Adam [31] optimizer was used with 0.0001 learning rate. Inception-V3 network was trained using Python 3.11.9 version with Tensorflow 2.17.0 [32] and Keras 3.5.0 [33]. A MobileNet-V1 [34] model was also trained with ROI images, it was observed that training accuracy and loss metrics were not optimal. Inception-V3 model was selected as the Convolutional Neural Network architecture.

Gesture recognition can be considered as an object recognition problem. An object recognition model, the Yolo-8 nano network, was trained on images from the synthetic dataset. Box regions were marked around the hands with gesture class labels, and the coordinates of boxes were obtained using the same tip point detection method mentioned in Inception-V3 network. Yolo-8n was also trained using five-fold cross validation. In each fold, 25% of the dataset (24,000 frames) was selected for validation, and 75% of the dataset was used for training (72,000 frames).

**Figure 8:**
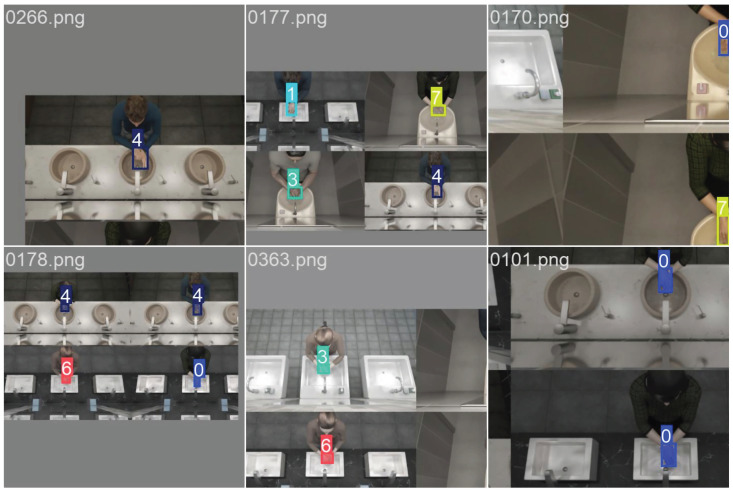
Automatically generated pre-training images of Yolo-8n (top row) and Yolo-8n segmentation (bottom row) networks. Boxes, labels and segmentations can be seen.

**Figure 9:**
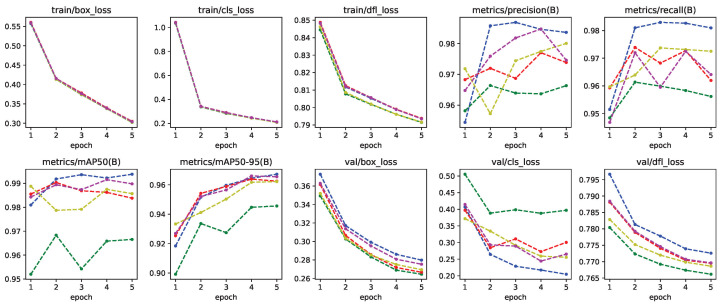
Training metrics for all five-fold cross validation models of Yolo-8n network.

Default parameters were used for Yolo-8n except for the flipud; fliplr was set to zero because of gestures being symmetrical to each other. Yolo-8n creates artificial variations in data pre-training and flipped images can alter the performance of the network in this situation. In Figure 8, images prepared by the Yolo-8n before training can be seen. Yolo-8n was trained for five epochs; more epochs were resulted in overfitting. Training metrics for this model can be seen in Figure 9.

**Figure 10:**
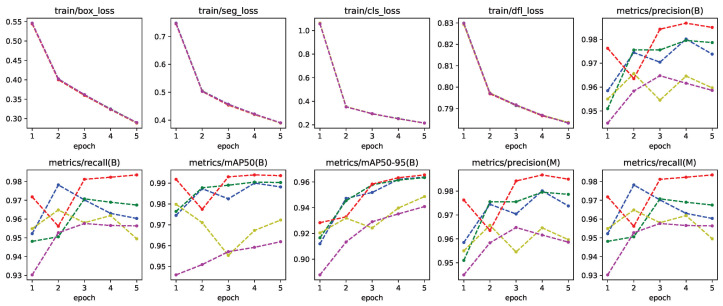
Training metrics for all five-fold cross validation models of Yolo-8n segmentation network.

Yolo-8n segmentation network was trained with rendered images using contour information obtained from the binary mask images in the dataset. Labels were created for segments corresponding to the gesture of the frame. This model was also trained using five-fold cross validation, using 25% for validation and 75% for training. Default parameters were used during training, with flipud and fliplr being set to zero similar to Yolo-8n. Training metrics for this model can be seen in Figure 10.

**Figure 11:**
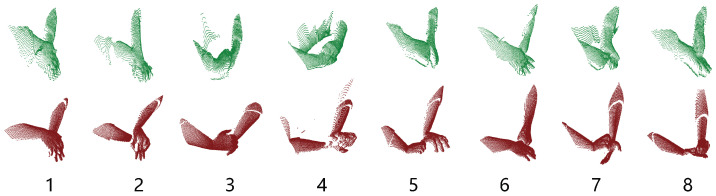
Comparison of real-world dataset point clouds (green) with the synthetic dataset point clouds (red). Gestures left to right: 1–8.

**Figure 12:**
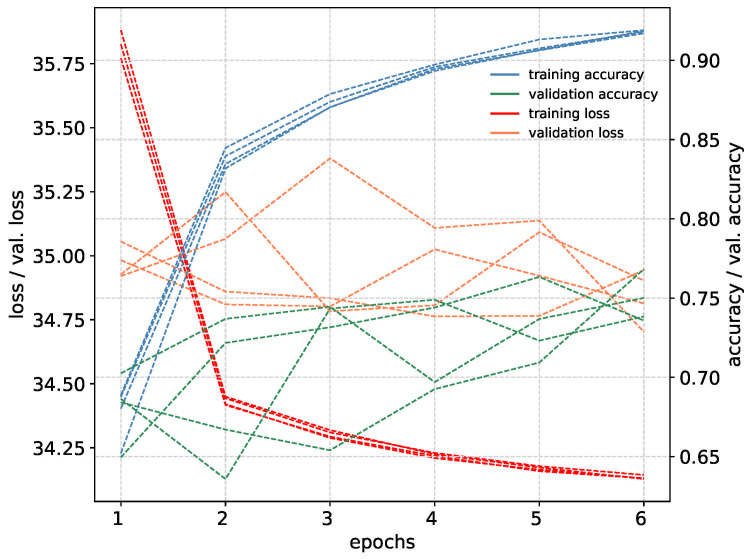
Training metrics for all five-fold cross validation models of PointNet network.

PointNet network can classify point clouds that are created from depth images. Depth ROI images were created using the same method used to create RGB ROI images. Binary mask images also correspond to the same coordinates in depth images. Using mask images, hand regions were cropped and 150 × 150 pixel images were created. Point clouds were created from these ROI images using Open3d [35]. To only include hand sections from point clouds, the depth value of the tip point pixel in depth ROI images were calculated, and a threshold of +3 and −1 in the depth value (Z axis) was used for selecting points. In total 512 points were uniformly sampled from each point cloud for network training. Because of normalized depth images from Blender, point clouds in 3D were formed very closely to each other in the Z axis. To overcome this, fx, fy parameters in Open3d were set to 7000 to increase the distance between points. This value was selected comparing the synthetic point cloud with the real-world point cloud using Open3d visualization. Both point clouds were added to the same scene and different values were tested to create the closest match between two. Depth scale parameter was set to 1000. Point clouds were centered around the origin using translation from Open3d functions. A comparison of synthetic dataset point clouds and real-world dataset point clouds can be seen in Figure 11. The network was trained with five-fold cross-validation for six epochs, using an Adam optimizer with 0.0001 learning rate. More epochs did not yield more accuracy during training. Training metrics can be seen in Figure 12.

## 4. Results

**Figure 13:**
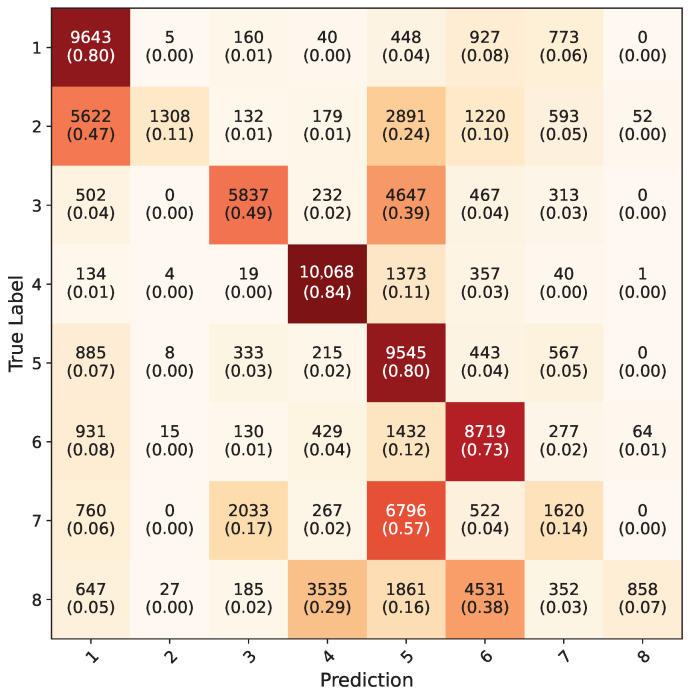
Average confusion matrix for pre-trained Inception-V3 models with real-world dataset, tested on synthetic dataset.

Trained models were tested on the real-world hand washing dataset from previous study [4]. In the previous study, the Inception-V3 model was trained on RGB ROI images. To have an insight about the similarity of the synthetic dataset to the real-world dataset, five-fold cross-validated pre-trained Inception-V3 models from previous study were tested on the synthetic dataset. Overall, an accuracy of 49.5% was acquired. The average confusion matrix for these models can be seen in Figure 13.

**Figure 14:**
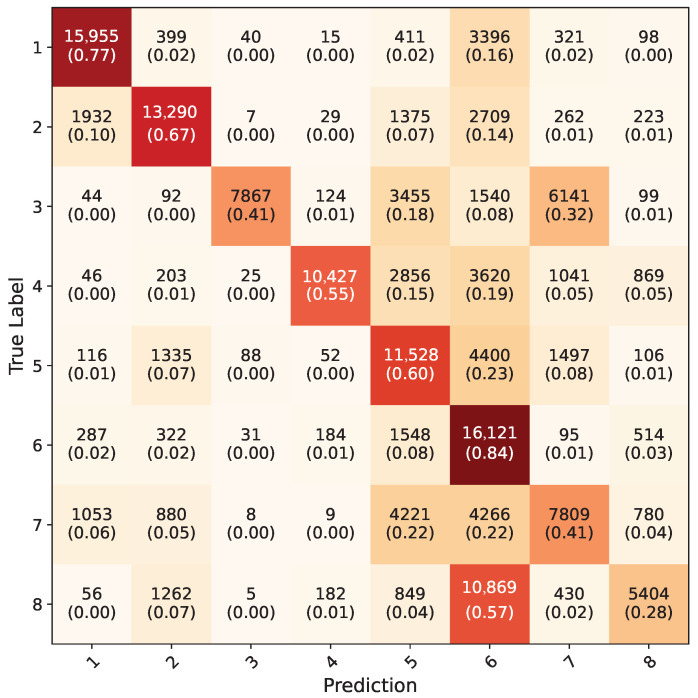
Average confusion matrix for all five-fold cross validation models of Inception-V3 network.

**Figure 15:**
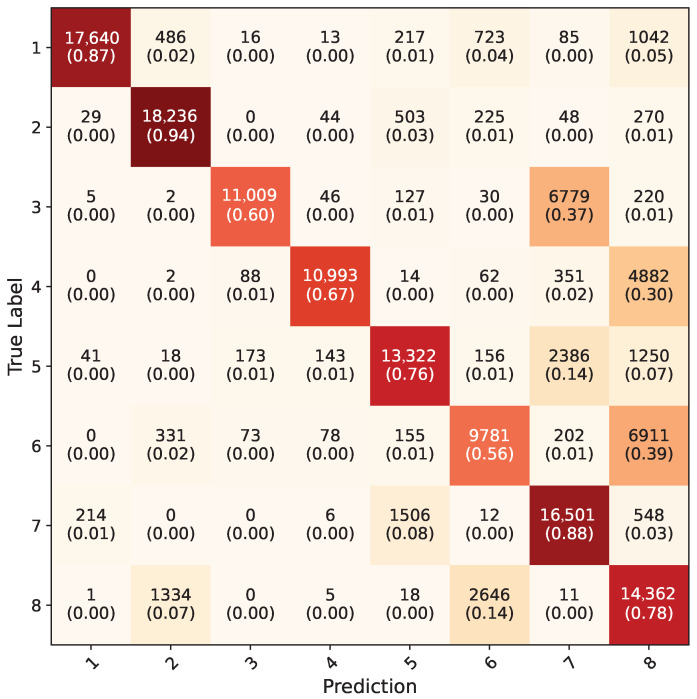
Average confusion matrix for all five-fold cross validation models of Yolo-8n network.

Inception-V3 five-fold cross validation models that were trained using the synthetic dataset were tested on RGB ROI images in the test dataset; the average confusion matrix of these five models is given in Figure 14. The inception models acquired a 56.9% overall accuracy for all the gestures. The testing of Yolo-8n five-fold cross validation models on the test dataset using uncropped RGB images resulted in a 76.3% overall accuracy; the average confusion matrix can be seen in Figure 15. During testing, the confidence value of this model was set to be 0.1 because all eight gestures consist of the same kinds of objects, which are hands. With this value, the frames with which all the five-fold validation models made a detection were counted and included in calculation of confusion matrix. Models successfully detected hands in 146,400 frames out of 154,683, acquiring a 94.5% accuracy. When frames were examined where any of the models could not detect, it was observed that they were faulty frames (all black, or frames which do not contain hands), which the test dataset contained in minuscule amounts.

**Figure 16:**
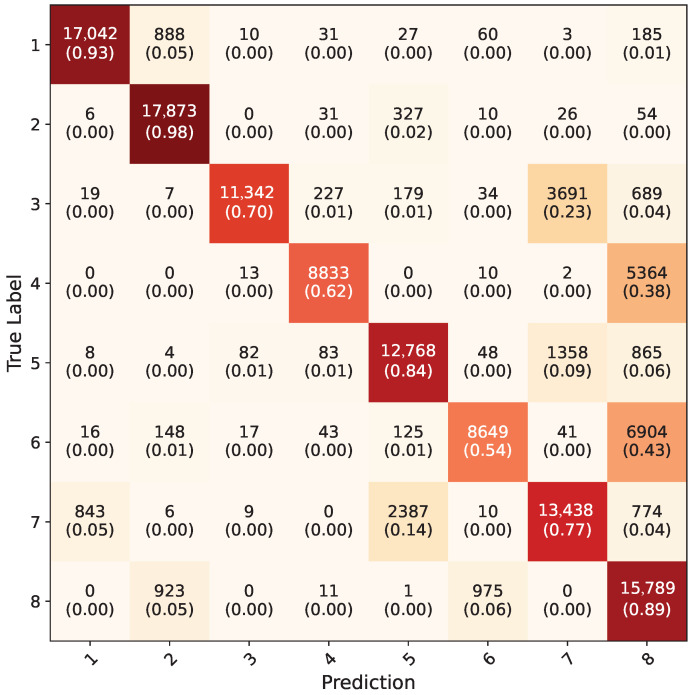
Average confusion matrix for all five-fold cross validation models of Yolo-8n segmentation network.

**Figure 17:**
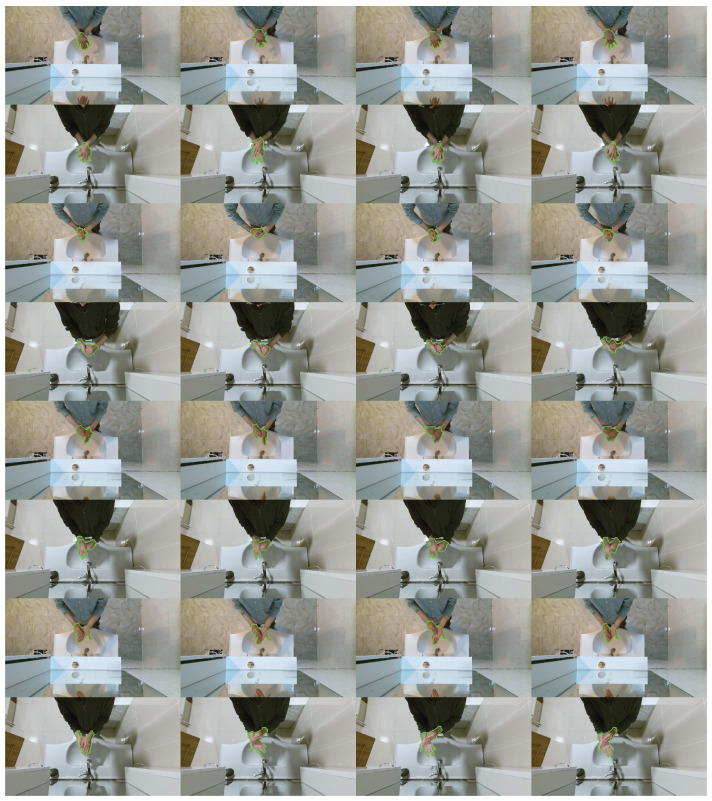
Example contour results of Yolo-8n segmentation model on random frames for each gesture in test dataset.

The average confusion matrix of the Yolo-8n segmentation network on uncropped RGB images from the test dataset can be seen in Figure 16. This model has acquired highest overall accuracy, with 79.3%. Like in the Yolo-8n object detection model, frames where all five-fold models detected the hand region were counted and included in calculation of the confusion matrix. In total, 133,334 frames out of 154,683 were counted, making the hand detection rate 86%. Undetected frames here were also mostly faulty frames in the test dataset. Because of the test dataset not having any segmentation labels originally, contour accuracy could not be measured directly. However, contours were drawn using OpenCV over the entire dataset and highly accurate results were observed. In Figure 17, the contour results of the Yolo-8n segmentation model on random frames from the test dataset for each gesture can be seen.

**Figure 18:**
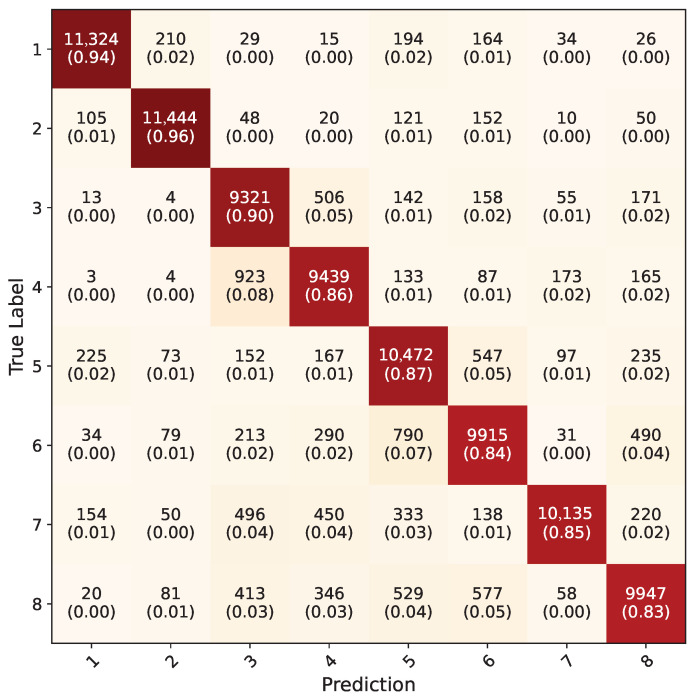
Average confusion matrix for all five-fold cross validation models of PointNet network on synthetic dataset.

The PointNet model, although performing well on the synthetic dataset (88% accuracy), showed lower performance on the real-world dataset, as it obtained a 26% overall accuracy. In Figure 18, the confusion matrix for the PointNet models on the synthetic dataset can be seen. Keras version of the PointNet was used [36]. Creating point clouds to represent real-world data can be challenging for several reasons. Blender’s default depth image value output range is limited. Normalizing depth data is a possibility in post processing settings, however this option also generates different normalized values for background isolated depth images and default scene depth images. Furthermore, matching the intrinsic and extrinsic matrices of the Kinect 2 with Blender may be needed, and a further study is required to replicate exact depth sensor. Furthermore, point cloud data for a scene in the real world can vary from one depth sensor to another. Particularly on the hand washing subject, the reason for low accuracy can be due to gestures being symmetrical and very similar to each other. Another reason may be empirically selecting fx, fy values to resemble similar point clouds to Kinect 2 point cloud data.

**Figure 19:**
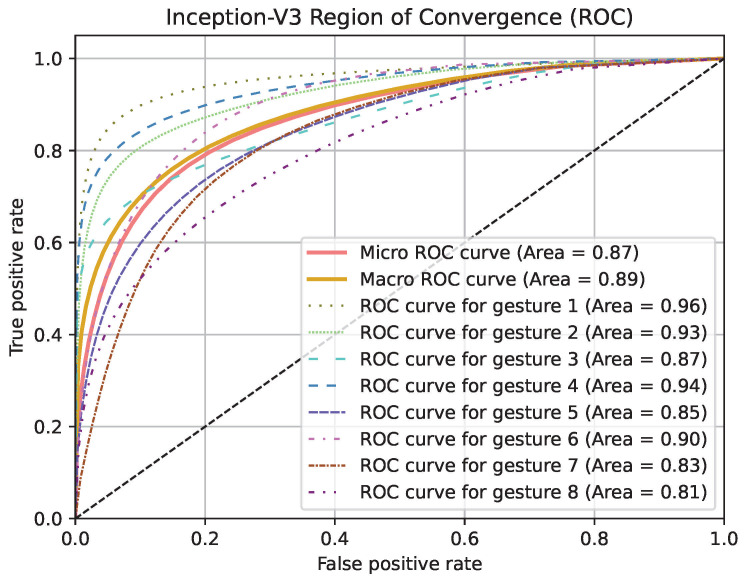
ROC curve graphic of Inception-V3 model for each gesture and overall.

**Figure 20:**
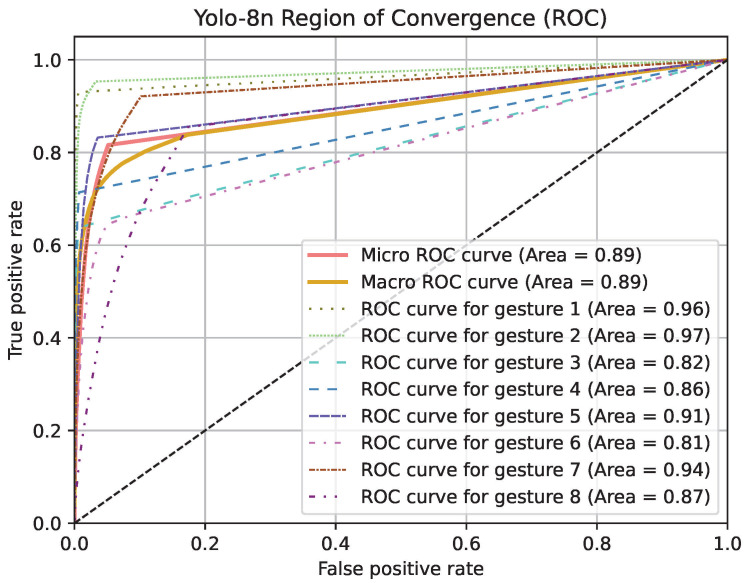
ROC curve graphic of Yolo-8n model for each gesture and overall.

**Figure 21:**
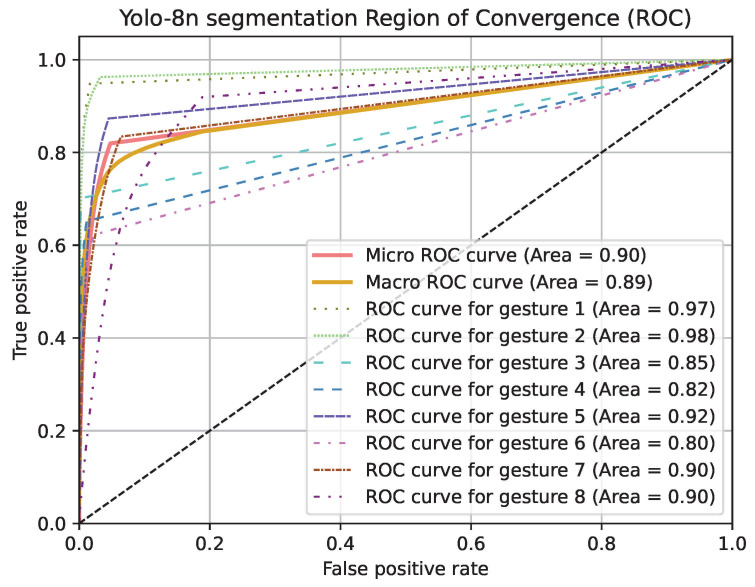
ROC curve graphic of Yolo-8n segmentation model for each gesture and overall.

Region of Convergence (ROC) curve graphics for models except PointNet can be seen in Figure 19, Figure 20 and Figure 21. Averaged sensitivity, specificity, F1 and ROC AUC values averaged for all gestures for all cross validation models of networks are given in Table 4. These values were calculated using Scikit-Learn 1.5.2 [37]. Overall accuracies by gesture for all models are given in Table 5.

Observing the results, the Yolo-8n segmentation model was the best performing among all. The highest accuracy was seen with the Yolo-8n segmentation model in gesture 2 with 98%. Lowest accuracy was seen with Inception-V3 model in gesture 8, with 28%.

A comparison of gesture count, data type and data amount of various vision-based hand washing recognition studies can be seen in Table 6.

## 5. Discussion

To overcome the difficulty of creating datasets for hand washing gesture evaluation, an open synthetic dataset was created in this research. On this dataset, Inception-V3, Yolo-8n, Yolo-8n segmentation and PointNet networks were trained and their performance of gesture classification was measured in a real-world dataset.

Results showed that, synthetic data have been useful for accurate classification. PointNet network trained with synthetic point clouds performed with lower accuracy than others, while Inception-V3 network acquired a 56.9% accuracy, Yolo8-n acquired 76.3% and Yolo-8n segmentation acquired 79.3% being the highest.

Observing the results, it can be concluded that the more realistic and varied a synthetic dataset is, the more successful it will be. Synthetic depth images can also be useful, as PointNet showed significant improvement over random decision. However, it can be said that it would benefit most if objects/gestures are varied and distinct enough. Current available data in this study may not be sufficient, more synthetic and real-world test data are required for further investigation. In addition, rendering software may need to be calibrated accordingly to produce synthetic depth images that resemble real depth sensors.

It is observed in proposed approach that in situations where creating a real-world dataset is difficult, using computer-generated imagery may be sufficient enough. Details about the dataset, source codes and sample test data will be available at [47]. Further research in this topic can benefit from the standardization of washing gestures and camera positions. Current datasets are not enough for benchmarking different methods, because of camera position and gesture differences. With standardization, sufficient amount of varied data can be obtained, thus the issue of biased datasets or overfitting can be eliminated. Computer vision-based models can also measure soap amount, using a separate neural network or raw image processing techniques. Hand rubbing evaluation/motion quality can be achieved using methods similar to optical flow analysis. Point clouds with color information can be beneficial for isolating only hand/arm segments, or specially trained point cloud segmentation models can be used for segmenting hands/arms in point clouds.

## Figures and Tables

**Table 1:** Approximate frame rendering time (seconds) for environments.

System	Environment 1	Environment 2	Environment 3	Environment 4
1	2–3	2–3	2.5–3.5	2.5–3.5
2	25–27	19–21	24–26	32–34
3	39–42	31–34	40–42	57–60

**Table 2:** Frame amounts for eight gestures in real-world test dataset.

Gestures	RGB and RGB ROI	Point Cloud
1	20.637	59.883
2	19.831	59.173
3	19.365	58.112
4	19.089	57.667
5	19.125	57.383
6	19.106	57.483
7	19.029	57.751
8	19.060	57.786
Total	155.242	465.238

**Table 3:** Inception-V3 training metrics.

	Accuracy	Loss	Validation Acc.	Validation Loss
Epoch 1	0.904	0.277	0.998	0.007
Epoch 2	0.994	0.023	0.997	0.010

**Table 4:** Additional statistics on results for each model, averaged for all five-fold cross validation models.

	Inception-V3	Yolo-8n	Yolo-8n Segmentation
Sensitivity	0.672	0.808	0.821
Specificity	0.927	0.965	0.967
F1 Macro	0.573	0.765	0.774
F1 Micro	0.569	0.762	0.772
ROC AUC	0.885	0.890	0.893

**Table 5:** Overall performance of all models by gesture and average.

Gestures	Inception-V3	Yolo-8n	Yolo-8n Segmentation
1	77%	87%	93%
2	67%	94%	98%
3	41%	60%	70%
4	55%	67%	62%
5	60%	76%	84%
6	84%	56%	54%
7	41%	88%	77%
8	28%	78%	89%
Average	56.9%	76.3%	79.3%

**Table 6:** Comparison of vision-based hand washing studies.

Study	Data Type	Gesture Count	Data Amount
Llorca et al. [38]	Color Frame	6	8.408 Frames
Xia et al. [39]	Color/Depth Frame	12	72 Videos
Dietz et al. [40]	Depth Frame	9	67.375 Frames
Zhong et al. [41]	Color Frame	7	2055 Videos
Ivanovs et al. [42]	Color Frame	7	309.315 Frames
Kim et al. [43]	Color Frame	4	47.249 Frames
Prakasa & Sugiarto [44]	Color Frame	6	1.647 Frames
Lulla et al. [11]	Color Frame	6	3.185 Videos
Q.Vo et al. [45]	Color Frame	7	731.147 Frames
Zhong et al. [46]	Color Frame	4	464 Videos
Xie et al. [10]	Color Frame	7	656 Videos
Haghpanah et al. [5]	Color Frame	9	1.100 Videos
Pepito et al. [9]	Color Frame	5	20.333 Frames
Ozakar & Gedikli [4]	Color/Depth Frame	8	155.242/465.238 Frames
Asif et al. [8]	Color Frame	4/7	451/656 Videos
Ju & Reibman [6]	Synthetic Color Frame	5	518.000 Frames
This Study	Synthetic Color/Depth Frame	8	96.000 Frames

## Data Availability

Dataset will be publicly available on: github.com/r-ozakar (accessed on 7 May 2025).
